# Mitochondrial and nuclear phylogenetic trees and divergence time estimations of Sulawesi endemic Adrianichthyidae

**DOI:** 10.1016/j.dib.2015.08.032

**Published:** 2015-09-03

**Authors:** Daniel F. Mokodongan, Kazunori Yamahira

**Affiliations:** Tropical Biosphere Research Center, University of the Ryukyus, Okinawa 903-0213, Japan

## Abstract

This data article is related to the research article entitled “Origin and intra-island diversification of Sulawesi endemic Adrianichthyidae” by Mokodongan and Yamahira [1]. In this data article, we present phylogenetic trees of Sulawesi adrianichthyids separately reconstructed using mitochondrial (cytochrome *b*: cyt *b* and NADH dehydrogenase subunit 2: ND2) and nuclear (*tyrosinase*) sequences. We also present Bayesian chronograms of Sulawesi adrianichthyids separately estimated using a substitution rate for cyt *b* and for ND2.

## Specifications table

**Table:** 


Subject area	Biology, Genetics and Genomics
More specific subject area	Phylogenetics and phylogenomics
Type of data	Phylogenetic trees, chronograms
How data was acquired	Phylogenies were acquired using maximum likelihood and Bayesian inference methods. Chronograms were acquired using Bayesian inference methods
Data format	Analyzed
Experimental factors	Phylogenies were estimated by raxMLGUI and MrBayes using mitochondrial (cyt *b* and ND2) and nuclear (*tyr*) sequences. Bayesian chronograms were estimated by BEAST using two different molecular clocks.
Experimental features	Codon-specific GTR+I+G models and a rapid bootstrap analysis of 1000 bootstrap replicates were used in raxmlGUI. MrBayes was run using ngen=3000000, samplefreq=100, and burnin=30000. BEAST analyses were performed using ngen=50000000, samplefreq=1000, burnin=12500000, and a substitution rate of 2.65% and 2.8% per My for cyt *b* and ND2, respectively.
Data source location	n/a
Data accessibility	With this article

## Value of the data

• Data on phylogenies separately estimated using mitochondrial and nuclear sequences enable researchers to examine how the topologies differ from each other.
• Data on phylogenies of Sulawesi adrianichthyids enable researchers to compare their diversification history with those of other Sulawesi taxa.
• Data on two alternative chronograms enable researchers to infer the possible ranges of time frames in the divergence events of Sulawesi taxa.

## 1 Data, experimental design, materials and methods

### 1.1 Phylogenetic trees of Sulawesi adrianichthyids

**Fig. 1:**
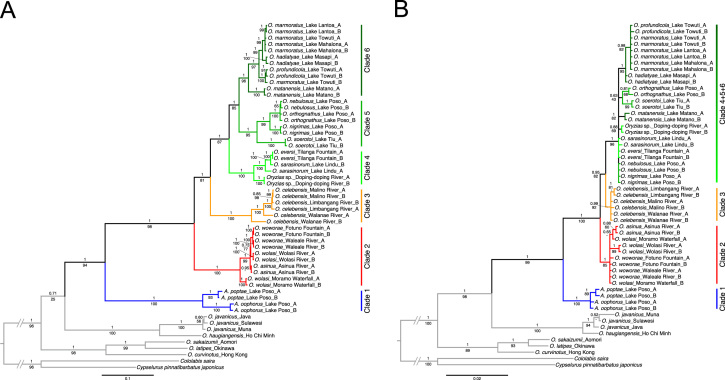
Bayesian inference phylogenies of Sulawesi adrianichthyids based on (A) mitochondrial sequences (cyt *b*: 1141 bp and ND2: 1046 bp) and (B) nuclear sequences (*tyr*: 1253 bp). Numbers on branches represent Bayesian posterior probabilities (top) and maximum likelihood bootstrap values (bottom). The scale bar indicates the number of substitutions per site. The major lineages of Sulawesi adrianichthyids are color coded.

The data presented here represent the phylogenetic trees of Sulawesi adrianichthyids separately reconstructed using mitochondrial (cytochrome *b*: cyt *b* and NADH dehydrogenase subunit 2: ND2) and nuclear (*tyrosinase: tyr*) sequences (Fig. 1A and B). The phylogenetic tree using concatenated sequences of the mitochondrial and nuclear sequences was presented in [1]. The data of the mitochondrial, nuclear, and combined phylogenetic trees in the Newick format are available as Supplementary data 1–3, respectively.

All of the sequence data used in this data article have been deposited into DNA Data Bank of Japan (DDBJ) under accession numbers LC51634–LC51792 for adrianichthyids and AP002932, AB569470, AB188743, and AB188744 for the two beloniform outgroups. They were aligned using ClustalW version 1.4 [2], and the alignment was later corrected by eye. The aligned sequences of the mitochondrial genes (cyt *b*: 1141 bp and ND2: 1046 bp), were concatenated into a single sequence (2187 bp). For the aligned sequence set of the nuclear gene (*tyr*), any site in intron 1 where a gap was found in any of the populations/species was removed, which resulted in a 1253-bp sequence (exon 1=809 bp, intron 1=269 bp, and exon 2=175 bp). Heterozygous sites in the *tyr* sequences (R, Y, M, K, W, and S) were replaced with N. The aligned mitochondrial and nuclear sequences were separately analyzed. Thereafter, the sequences were combined into a single 3440-bp concatenated alignment, and analyses were performed on the combined sequences.

Phylogenies were estimated by maximum likelihood (ML) and Bayesian inference (BI) methods. ML analyses were performed by raxmlGUI version 1.31 [3] using codon-specific GTR+I+G models for each sequence set (however, codon positions were not partitioned for *tyr* intron 1), where a rapid bootstrap analysis of 1000 bootstrap replicates was conducted. BI analyses were conducted with MrBayes version 3.2.4 [4]. Appropriate substitution models were separately determined for cyt *b*, ND2, *tyr* exon 1, and *tyr* exon 2 codon positions using jModelTest version 2.1.7 [5] based on the Akaike Information Criterion (AIC) (Table 2 in [1]). Similarly, the GTR+G model was chosen for *tyr* intron 1 without separating codon positions. Analyses were run using the following settings: ngen=3000000, samplefreq=100, and burnin=30000.

### 1.2 Bayesian chronograms of Sulawesi adrianichthyids

**Fig. 2:**
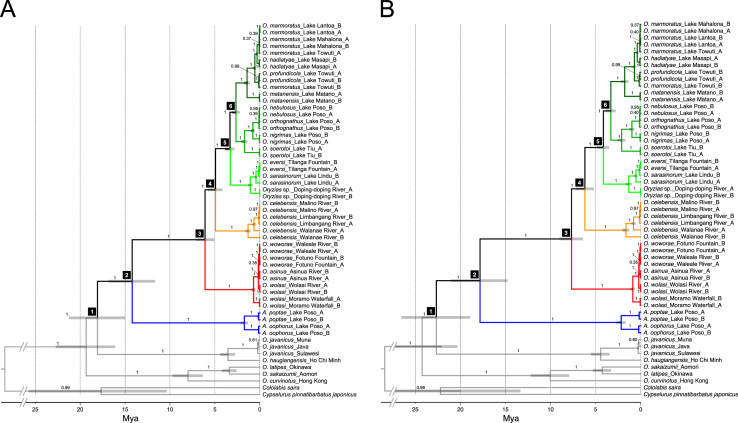
Bayesian chronograms of Sulawesi adrianichthyids based on the mitochondrial sequences (cyt *b*: 1141 bp and ND2: 1046 bp) using a substitution rate of (A) 2.65% per My for cyt *b* and (B) 2.8% per My for ND2. Numbers on branches are Bayesian posterior probabilities. Bars represent 95% high posterior density. The major lineages of Sulawesi adrianichthyids are color coded.

The data presented here represent the Bayesian chronograms of Sulawesi adrianichthyids separately estimated using a substitution rate for cyt *b* and for ND2 (Fig. 2A and B). The data of the two chronograms in the Newick format are available as Supplementary data 4 and 5.

Lognormal relaxed clock analyses were performed in BEAST version 1.8.1 [6] on the 2187-bp mitochondrial sequence dataset. Appropriate substitution models were determined for each gene (GTR+I+G for both cyt *b* and ND2) using the jModelTest. Analysis was run by partitioning codon positions and using the following settings: birth-death process, ngen=50000000, samplefreq=1000, and burnin=12500000. Analysis was run separately specifying substitution rates as (1) 2.65% per My for cyt *b* and “estimate” for ND2 and as (2) 2.8% per My for ND2 and “estimate” for cyt *b*. Both of the clocks were based on previous studies on divergence time estimations of Adrinichthyidae [7], [8].
